# Heparan sulfate proteoglycans and cancer

**DOI:** 10.1054/bjoc.2001.2054

**Published:** 2001-10

**Authors:** F H Blackhall, C L R Merry, E J Davies, G C Jayson

**Affiliations:** Cancer Research Campaign Department of Medical Oncology, Christie Hospital and Paterson Institute for Cancer Research, Wilmslow Road, Withington, Manchester, M20 4BX, UK

**Keywords:** heparan sulfate, proteoglycans, cancer

## Abstract

Heparan sulfate proteoglycans (HSPGs) are widely distributed in mammalian tissues and involved in a number of processes related to malignancy. They are composed of a core protein to which chains of the glycosaminoglycan, heparan sulfate (HS), are attached. The existence of various classes of core protein, in addition to highly polymorphic HS chains, creates a superfamily of macromolecules with considerable diversity of structure and function. HSPGs interact with many proteins including growth factors, chemokines and structural proteins of the extracellular matrix to influence cell growth, differentiation, and the cellular response to the environment. The recent identification of two inherited syndromes that are associated with an increased cancer risk, and caused by mutations in HSPG-related genes, has intensified interest in these molecules. This review describes our current understanding of HSPGs in cancer and highlights new possibilities for therapeutic control. © 2001 Cancer Research Campaign  http://www.bjcancer.com


*British Journal of Cancer* (2001) **85**(8), 1094–1098
© 2001 Cancer Research Campaign
doi: 10.1054/ bjoc.2001.2054, available online at http://www.idealibrary.com on IDEAL
http://www.bjcancer.com

Review

### Heparan sulfate proteoglycans and cancer

**FH Blackhall, CLR Merry, EJ Davies and GC Jayson**

Cancer Research Campaign Department of Medical Oncology, Christie Hospital and Paterson Institute for Cancer Research, Wilmslow Road, Withington, Manchester M20 4BX, UK

**Summary** Heparan sulfate proteoglycans (HSPGs) are widely distributed in mammalian tissues and involved in a number of processes related to malignancy. They are composed of a core protein to which chains of the glycosaminoglycan, heparan sulfate (HS), are attached. The existence of various classes of core protein, in addition to highly polymorphic HS chains, creates a superfamily of macromolecules with considerable diversity of structure and function. HSPGs interact with many proteins including growth factors, chemokines and structural proteins of the extracellular matrix to influence cell growth, differentiation, and the cellular response to the environment. The recent identification of two inherited syndromes that are associated with an increased cancer risk, and caused by mutations in HSPG-related genes, has intensified interest in these molecules. This review describes our current understanding of HSPGs in cancer and highlights new possibilities for therapeutic control. © 2001 Cancer Research Campaign http://www.bjcancer.com

**Keywords**: heparan sulfate; proteoglycans; cancer

Heparan sulfate proteoglycans (HSPGs) are common constituents of cell surfaces and the extracellular matrix (ECM), including basement membranes (see Bernfield et al, 1999; Gallagher and Lyon, 2000 for reviews). There is now ample evidence that HSPGs are essential for normal cell growth and development. This came initially from biochemical and cell culture studies that suggested a key co-receptor function for HSPGs in mediating cell responses to growth factors and chemokines. More recently, genetic studies in *Drosophila* and mice (Selleck, 2000) and identification of the genes responsible for two inherited diseases in man, the Simpson Golabi Behmel syndrome (SGBS) and hereditary multipleexostoses (HME), have demonstrated that alterations in HSPG synthesis lead to phenotypic changes indicative of aberrant control of cell growth, differentiation, organogenesis, and bone formation. In addition, a tumour suppressor function has been attributed to the genes causing SGBS and HME because affected individuals are at increased risk of cancer.

### HEPARAN SULFATE PROTEOGLYCANS AND CELL REGULATION

HSPGs comprise a protein core to which chains of the glycosaminoglycan (GAG), heparan sulfate (HS) are covalently attached during post-translational modification (Figure 1). At the cell surface, the two major families of HSPG are the transmembrane syndecans (S) and the GPI-anchored glypicans (Gpc) (Bernfield et al, 1999). The HSPGs found in the ECM (perlecan, agrin, collagen XVIII) are large modular proteins, that contribute to the structure, hydration and permeability of the matrix. Functions attributed to both cell membrane and ECM HSPGs are varied. They sequester growth factors and provide protection from proteolytic degradation, enhance formation of receptor–ligand signalling complexes and direct ligands into the cell for degradation or recycling (see Bernfield et al, 1999; Gallagher and Lyon, 2000). The significance of HSPGs in cell regulation is further emphasised by the presence of HS-binding domains in cell adhesion proteins of the ECM, such as fibronectin. The concerted action of cell surface integrins and HSPGs (specifically the syndecans) direct cell attachment to these ECM substrates, leading to the formation of focal adhesions (Bernfield et al, 1999 and references therein). The capacity of HSPGs to interact with both the matrix architecture and soluble ligands (Table 1) defines a unique combination of properties that enables normal cells to sense, and respond to, controlling influences in their environment. Cancer cells employ various mechanisms to exploit these properties and gain a survival advantage.

*Received 6 February 2001*
*Revised 25 June 2001*
*Accepted 29 June 2001*

*Correspondence to:* FH Blackhall

### HSPGs IN CANCER

### Altered HSPG expression

HSPG expression is developmentally regulated and altered in various pathophysiological processes, including cancer. The expression patterns are believed to mirror those of ligands that require HSPGs to elicit their cellular responses. For example, Glypican 1 expression is up-regulated in pancreatic cancer cells and surrounding fibroblasts, and the mitogenic response of pancreatic cancer cells to bFGF and HB-epidermal growth factor is abrogated by anti-sense attenuation of this HSPG (Kleef et al, 1998). Similarly, perlecan expression is up-regulated at sites of active angiogenesis, and the angiogenic affects of bFGF are suppressed by experimental down-regulation of perlecan (Sharma et al, 1998).

### HSPG catabolism yields bioactive products

The breakdown products of HSPGs exhibit distinct physiological roles. Syndecans are shed from the cell surface as soluble molecules (ectodomains) that regulate protease and GF activity. For example, in contrast to their cell surface precursors, S1 ectodomains inhibit bFGF signalling (Bernfield et al, 1999). This change in function is believed to ‘fine tune’ GFs recruited for

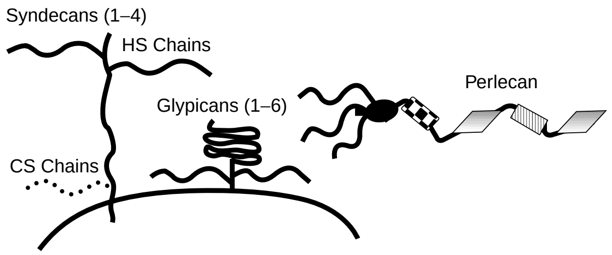

**Figure 1** Classification and structure of HSPGs. 4 mammalian syndecan isoforms (S1–4) are encoded by separate genes. These transmembrane proteins adopt highly extended conformations, have short cytoplasmic tails and HS attachment sites are clustered near the N-terminus in the ecotodomain. S1, 2 and 4 also carry chondroitin sulfate (CS) side chains in addition to HS. The syndecan core proteins are least homologous in the ectodomains although the HS attachment sites are highly conserved. Discrete genes have been described for 6 glypican core proteins (Gpc1–6). These core proteins are extracellular globular glycoproteins that attach to the cell membrane via a glycosylphosphatidylinositol (GPI) anchor and HS chains are positioned close to the cell membrane. The ECM-resident perlecan comprises a large multidomain, 400 kD core protein to which several HS chains attach to a terminal globule tissue repair and remodelling. Enhanced shedding of S1 is observed in cancer but its role is poorly understood (Stanley et al, 1999). An intriguing property of S1 ectodomains is that they inhibit growth of various cancer cell lines in vitro and S1 ectodomains shed from myeloma cells induce apoptosis of myeloma allografts in SCID mice (Dhodapkar et al, 1998). In clinical studies of multiple myeloma serum S1 is an independent prognostic indicator with ‘high’ levels conferring an adverse prognosis (Seidel et al, 2000).

### Heparanase mediates angiogenesis, tumour invasion and metastasis

The heparanase enzymes degrade HS chains and are believed to play a major role in tumour progression. They are present in multiple normal and malignant cell types, but have proved particularly difficult to characterise. A range of molecular weights for heparanase has been reported, suggesting a family of distinct enzymes. This theory was initially challenged by the recent cloning of a single heparanase gene (*HPA1*) encoding identical enzymes in human hepatoma, placenta and platelets (Vlodavsky et al, 1999). However, a second gene (*HPA2*) with significant homology to HPA1, that gives rise to 3 proteins by alternative splicing has subsequently been reported (McKenzie et al, 2000). Their functions have not been fully characterised but, interestingly, the tissue distribution and cellular localisation of HPA2 appears distinct from HPA1 suggesting different roles in HS catabolism.

In experimental models of cancer increased heparanase production correlates with enhanced metastatic potential. Heparanase activity has also been detected in the sera and urine of cancer patients although the prognostic/diagnostic significance of this has not been determined (Vlodavsky et al, 1999). Proteases (such as the MMPs) and heparanases are believed to cooperate in the degradation of the ECM and basement membranes. Interestingly, heparanase reverses the inhibitory activity of shed S1 ectodomains by liberating fragments of HS that activate bFGF (Bernfield et al, 1999). In contrast to the MMPs, only a few heparanases have been identified to date, suggesting that heparanase may be particularly amenable to therapeutic manipulation. Cancer cells can therefore manipulate HSPGs via various mechanisms. In addition, for some HSPGs a tumour suppressor (TSP) role has been proposed.

**Table 1** Heparin-binding proteins of the tumour microenvironment

| |
|---|
| Antithrombin III |
| Collagens |
| Elastase |
| Endostatin |
| Fibroblast growth factors including bFGF, aFGF, KGF |
| Fibrin |
| Fibronectin |
| Granulocyte-macrophage colony stimulating factor |
| Hepatocyte growth factor/scatter factor |
| Heparin-binding epidermal growth factor (HB-EGF) |
| Hyaluronidase |
| Insulin-like growth factors |
| Interferon-γ |
| Interleukin 3,8 |
| Laminin |
| L-selectin |
| Midkine |
| Neural cell adhesion molecule |
| Platelet-derived growth factor |
| Platelet factor 4 |
| Superoxide dismutase |
| Thrombospondin |
| Transforming growth factor β |
| Tumour necrosis factor |
| Urokinase |
| Vascular endothelial cell growth factor |

(Adapted from Bernfield, 1999).

### A novel family of tumour suppressors?

In experimental studies of malignant transformation, S1 expression is associated with maintenance of epithelial morphology, anchorage-dependent growth and inhibition of invasiveness (see Inki and Jalkanen 1996 for review). S1 is down-regulated in many epithelial cancers and in premalignant lesions of the oral mucosa and uterine cervix, its loss is an early genetic event contributing to tumour progression. Loss of S1 also correlates with reduced survival in squamous cell carcinoma of the head and neck, laryngeal cancer (Inki and Jalkanen, 1996) and malignant mesothelioma (Kumar-Singh et al, 1998); and higher metastatic potential in hepatocellular cancer (Matsumoto et al, 1997).

However, S1 also exhibits tumour promoter function. For example, S1 is essential for wnt-1 induced tumorigenesis of the mouse mammary gland (Alexander et al, 2000) and promotes formation of metastases in mouse lung squamous carcinoma cells (Hirabayashi et al, 1998). Enhanced S1 expression is observed in pancreatic cancer (Conejo et al, 2000) and the most invasive cells in ovarian cancer (J Davies–unpublished observation). This ‘dual role’ of S1 may reflect tissue-specific HSPG function. There is evidence for this in *Drosophila*, where the glypican homologue dally selectively participates with several GF signalling pathways in a tissue specific manner (Selleck, 2000 for review). The rare Simpson–Golabi–Behmel syndrome (SGBS) provides compelling evidence that an HSPG can function as a tumour suppressor.

### Glypican 3 – a negative regulator of cell growth

SGBS is an X-linked syndrome characterised by pre- and post-natal overgrowth and an increased frequency of embryonal tumours such as Wilm’s tumour of the kidney and neuroblastoma. Affected males

© 2001 Cancer Research Campaign 

can reach heights of two metres and often exhibit features including congenital heart defects and dysplastic kidneys. The syndrome is caused by mutation of the glypican 3 gene (GPC3) (Pilia et al, 1996) and is the first inherited disorder known to involve an HSPG. The developmental abnormalities associated with SGBS provide confirmation that GPC3 is a critical regulator of tissue growth and morphogenesis. It is believed that defects in GF signalling pathways due to loss of function of GPC3 (Veugelers et al, 2000) account for the abnormalities in SGBS, and possible candidates include insulin-like growth factor 2 (Pilia et al, 1996) and bone morphogenetic protein 4 (Paine-Saunders et al, 2000).

A TSP role for GPC3 has been proposed due to the incidence of embryonal tumours in SGBS, and the ability of GPC3 to induce cell-line-specific apoptosis in rat metastatic melanoma and human breast cancer MCF7 cells (Gonzalez et al, 1998). The mechanism of apoptosis is unclear, but is independent of glycosylation which, uncharacteristically for an HSPG (see next section), suggests a direct interaction between a ligand and the core protein rather than the HS chain. Location of a TSP on the X chromosome is intriguing since men, with only one copy, would be more vulnerable to tumour formation. However, GPC3 expression becomes restricted to the ovary and colon in adult tissues, and GPC3 may act as a negative regulator of growth exclusive to female tissues such as the ovary in vivo (Lin et al, 1999). Indeed, the chromosomal region encompassing the *GPC3* gene on Xq26 is deleted in approximately 30% of patients with sporadic ovarian cancer (Choi et al, 1997). Correspondingly, in a screen of ovarian cancer cell lines employing a candidate gene approach, no GPC3 mutations were identified, but transcriptional silencing by hypermethylation of the GPC3 promoter was detected in 30% of cell lines (Lin et al, 1999). Interestingly, the same mechanism (of loss) has been identified in a proportion of rat and human malignant mesothelioma cell lines and tumours (Murthy et al, 2000). Consistent with a TSP role, ectopic transfection of GPC3 inhibits cell growth in ovarian cancer cells that have lost endogenous GPC3 expression (Lin et al, 1999).

### Glycosylation – a major determinant of HSPG function

The ability of HSPGs to bind with high specificity to a wide range of diverse ligands is rooted in the structure of the HS chains (Gallagher and Lyon, 2000). A complicated biosynthetic machinery, that is not yet fully understood, enables cells to glycosylate core proteins for their own ligand-binding requirements (Figure 2). Mature HS is composed of hypervariable sulfated domains (S-domains) separated by regions of low sulfation (N-acetylated regions). This unique molecular design encodes specific recognition sites for multiple HB ligands. For example, antithrombin III recognises a unique pentasaccharide sequence containing a rare glucosamine 3-O-sulfate (Lindahl et al, 1998). This sequence is the basis for the clinical efficacy of the anticoagulant heparin and is thought to occur in the boundary between an S-domain and N-acetylated region. In contrast, bFGF binds with high affinity to S-domains characterised by a repeating disaccharide sequence $[\text{IdoA2S-GlcNS}]_5$ (Gallagher and Lyon, 2000). However, the additional presence of 6-O-sulfated GlcNS within this sequence is necessary for activation of bFGF. In turn, sequences that lack 6-O-sulfation are inhibitory and the cellular response to bFGF in vivo may ultimately depend on the balance of sequence presented (Pye et al, 1998). Although heparin is often used experimentally as a surrogate for HS, it lacks both the structural complexity and the N-acetylated regions. The function of these regions in vivo is uncertain but is likely to include the presentation

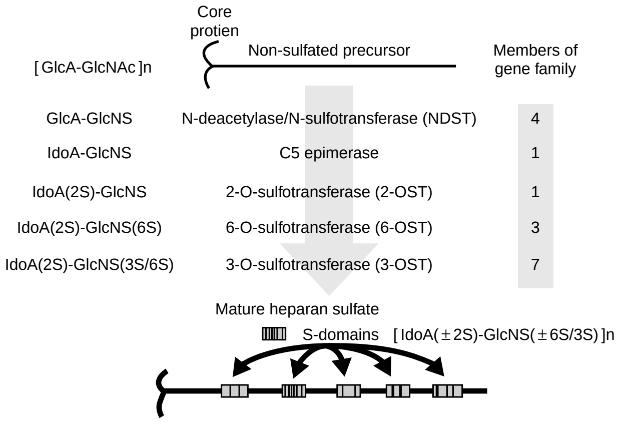

**Figure 2** A simplified scheme of heparan sulfate synthesis (see Lindahl 1998 for review). Heparan polymers comprise 50–200 N-acetylglucosamine-glucuronic acid (GlcNAc α1, 4 GlcA) repeats attached to core proteins at Ser-Gly repeat sequences. Transformation of heparan to HS is initiated by the N-deacetylase/N-sulfotransferase (NDST) enzymes, that modify a proportion (usually 40–50%) of GlcNAc to N-sulfoglucosamine (GlcNS). This, and the following modifications are non-random, interdependent and quantitatively incomplete. Uronate epimerase converts GlcA adjacent to GlcNS to the C-5 epimer, iduronate (IdoA). O-sulfotransferases act on resulting GlcNS/IdoA rich domains, beginning with HS-2OST which transfers sulfate to C-2 of the IdoA residues. Subsequently, 6-O and 3-O sulfotransferases (6- and 3-OSTs) complete the heparan-HS transition by transferring sulfate groups to C-6 or C-3 of the amino sugars. Members of the gene families recognise subtly different disaccharide substrates to create microstructurally heterogeneous sulfated (S) domains of S-domains in the correct orientation to dimeric and oligomeric ligands such as platelet factor 4 and transforming growth factor β that bind more than one S-domain simultaneously (Lyon and Gallagher, 2000).

The controlled variability of HS biosynthesis that is vital for the co-ordination of appropriate responses to pericellular ligands, may also be 'hijacked' by cancer cells. Structural alterations of HS are observed in various cell models of transformation (Jayson et al, 1998), lung cancer (Nackaerts et al, 1997) and human primary liver tumour sections (Matsumoto et al, 1997). Hereditary multiple exostosis (HME) provides the first direct evidence linking aberrant HS structure to tumorigenesis.

### Heparan sulfate biosynthesis and cancer

HME is an autosomal dominant disorder (estimated frequency 1:50 000) characterised by the presence of multiple bony outgrowths (exostoses) arising from the juxtaepiphyseal regions of long bones (Figure 3). The initial application of genetic linkage to HME families revealed this to be a multigenic disorder with 3 loci on chromosomes 8q24 (EXT1), 11p11-13 (EXT2) and 19p (EXT3). The phenotype of HME is identical for each locus, with the majority of cases attributed to mutations in EXT1 and 2. Additional gene family members not yet associated with disease include 3 EXT-like genes (*EXTL1*, *EXTL2*/*EXTR2*, *EXTL3*/*EXTR1*) (see Stickens and Evans, 1998 for review). In at least 2% of HME patients, exostoses undergo malignant transformation into chondrosarcomas or osteosarcomas (Hennekam, 1991). Both sporadic and exostosis-derived chondrosarcomas show loss of allelic heterozygosity for markers around the EXT 1 and EXT 2 loci suggesting a tumour suppressor (TSP) gene model (Hecht et al, 1995; Raskind et al, 1995).

The functions of EXT family members were discovered by chance during investigations of genes involved in GAG biosynthesis and could not have been anticipated (McCormick et al, 1998). EXT1

*© 2001 Cancer Research Campaign*

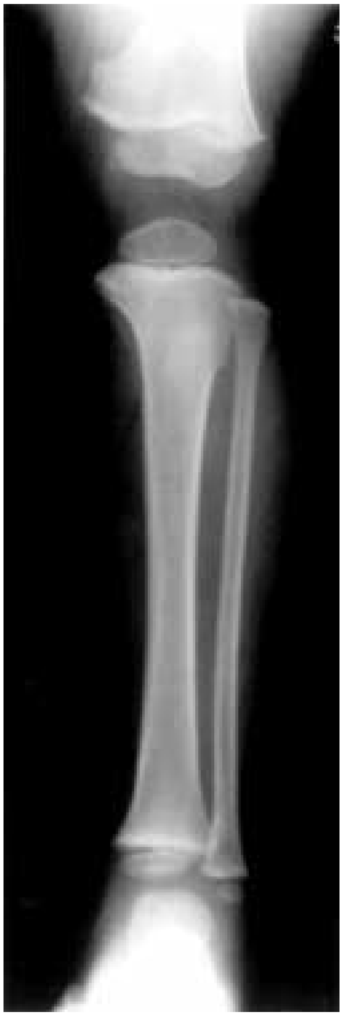

**Figure 3** Radiograph of an exostosis arising from the juxtaepiphyseal region of the femur in a 2 year old. Exostoses are benign osteochondromas that are at risk of malignant transformation. Photograph from Dr J Clayton Smith, Department of Medical Genetics, St Mary's Hospital, Manchester, with kind permission and EXT2, encode an enzyme (GlcA/GlcNAc transferase) required for chain elongation and synthesis of HS (Lind et al, 1998). EXTL2 encodes an $\alpha$1,4-N-acetylhexosaminyltransferase that initiates HS synthesis (Kitigawa et al, 1999). It is not implicated in HME, but has been assigned to a chromosome region (1p11–p12) that is frequently rearranged in sporadic cancer. Although the function of *EXTL3* is unknown, it is a candidate gene for the breast cancer locus on chromosome 8p12–p22 and is mutated in 25% of early colorectal carcinoma cell lines (Arai et al, 1999). In HME HS is assumed to regulate a critical signalling pathway(s) in bone formation. In mice, *EXT1* and *EXT2* become restricted to skeletal elements during embryonic development and their expression overlaps with Indian hedgehog, which controls chondrocyte maturation (Stickens et al, 2000). The HME story has fuelled interest in HS biosynthesis but also provided several puzzles. In particular, why is the disease phenotype restricted to bone, given the ubiquitous distribution of HS on cell surfaces and in the ECM? One possibility is that currently unidentified genes may substitute for *EXT1* and *EXT2* functions in non-osteogenic tissues (see below).

The contribution of other enzymes involved in HS biosynthesis to tumorigenesis has not been determined. Like the EXTs, the NDSTs, 6OSTs and 3OSTs (Figure 2) belong to multigene families (Selleck, 2000 and references therein). At least 4 distinct mammalian isoforms of NDST, 3 6OSTs and 7 3OSTs have been identified and shown to exhibit tissue-specific expression patterns. Preliminary studies have demonstrated a correlation between HS structure and isoform expression (Habuchi et al, 2000). The possibility of differential isoform expression in tumour tissues suggests the potential for selective manipulation of signalling pathways for therapeutic gain. For example, wild-type CHO cells spontaneously form tumours when injected subcutaneously into athymic mice, and inhibition of 2-O-sulfation of HS abolishes bFGF binding resulting in smaller tumours that are poorly vascularised (Esko, 1999).

### RESEARCH TOOLS

Progress in our understanding of HSPGs has been hampered by the lack of suitable tools to examine structure activity relationships but recent years have witnessed significant advances in the availability of such analytical techniques. Developmental genetic studies, particularly in *Drosophila*, and manipulation of genes encoding biosynthetic enzymes for HS have accelerated functional analyses of HSPGs. The availability of recombinant exoenzymes, used in the treatment of inherited mucopolysaccharidoses has inspired various techniques sensitive enough to reveal the exact order and composition of monosaccharides within an HS sequence of interest akin to DNA or peptide sequencing (Merry et al, 1999). Immunohistochemistry and in situ hybridisation studies of HSPG core proteins within human tumour sections have generated expression maps to correlate with clinical data. However, structural information about HS in situ has been limited due to the poor immunogenicity of HS and difficulty in raising antibodies. The application of phage display technology has generated new antibodies that may be capable of discriminating distinct HS epitopes (van Kuppevelt et al, 1998). One caveat in the interpretation of immunohistochemical studies of HSPGs is the influence of cellular origin and context on the structure and function of these molecules. To address this, a novel functional assay has been designed to detect the ability of HSPGs to form signalling complexes in situ (Chang et al, 2000). Tissues of interest are preincubated with bFGF (that binds to HS) then exposed to a soluble cognate-signalling receptor (FR) linked to alkaline phosphatase (AP). FR-AP is detectable if the HSPG–bFGF interaction favours recuritment of the soluble receptor into a trimolecular (signalling) complex. This technique has revealed that extracellular matrix HSPGs such as perlecan can bind bFGF strongly, but fail to incorporate FR-AP, suggesting an inhibitory role. Preliminary findings using this technique in ovarian cancer sections also suggest that some bFGF–HS complexes are biologically inactive (J Davies, unpublished observation). The assay is suitable for adaptation to recognise other signalling pathways e.g. VEGF, and isolation of HSPG isoforms present in active versus inactive signalling complexes should provide novel insights into growth factor regulation by HSPGs in different tissues.

### GLYCO-ONCOLOGY: THE CHALLENGE

HSPGs play pivotal roles in cellular control, co-ordinating and directing appropriate responses to multiple ligands. The natural mechanisms regulating their activities may be exploited by cancer cells that liberate varying cocktails of growth factors, cytokines, proteases and heparanase(s). Furthermore, evidence from 2 inherited human syndromes supports a direct role for HSPGs in tumorigenesis. The clinical success of heparin has prompted the development of

© 2001 Cancer Research Campaign

HSPG 'mimics' to antagonise HB growth factors and heparanase(s). However, in view of the tissue-specific and multifaceted interactions of these molecules, caution is required to avoid promoting unwanted ligand interactions caused by HS(PG) mimics that lack sufficient specificity. The present challenge is to combine the recent advances in genetic, molecular, biochemical and immunological tools to further unravel the roles of HSPGs in cancer, and to characterise novel targets for 'safe' therapeutic control. To this end, the biosynthetic machinery for HS may hold the key to selectively modulate critical HSPG-dependent signalling pathways.

### ACKNOWLEDGEMENTS

The authors would like to thank Professor JT Gallagher for helpful comments. Fiona Blackhall is funded by a Cancer Research Campaign Research Fellowship for a Clinician.

### REFERENCES

Alexander CM, Reichsman F, Hinkes MT, Lincecum J, Becker KA, Cumberledge S and Bernfield M (2000) Syndecan-1 is required for Wnt-1-induced mammary tumorigenesis in mice. *Nat Genet* **25**: 329–332

Arai T, Akiyama Y, Nagasaki H, Murase N, Okabe S, Ikeuchi T, Saito K, Iwai T and Yuasa Y (1999) EXTL3/EXTR1 alterations in colorectal cancer cell lines. *Int J Oncol* **15**: 915–919

Aviezer D, Iozzo RV, Noonan DM and Yayon A (1997) Suppression of autocrine and paracrine functions of basic fibroblast growth factor by stable expression of perlecan antisense cDNA. *Mol Cell Biol* **17**: 1938–1946

Bernfield M, Gotte M, Park PW, Reizes O, Fitzgerald ML, Lincecum J and Zako M (1999) Functions of cell surface heparan sulphate proteoglycans. *Ann Rev Biochem* **68**: 719–777

Chang Z, Meyer K, Rapraeger A and Friedl A (2000) Differential ability of heparan sulfate proteoglycans to assemble the fibroblast growth factor receptor complex *in situ*. *FASEB J* **14**: 137–144

Choi C, Cho S, Horikawa I, Berchuck A, Wang N, Cedrone E, Jhung SW, Lee JB, Kerr J, Chenevix-Trench G, Kim S, Barrett JC and Koi M (1997) Loss of heterozygosity at chromosome segment Xq25–26.1 in advanced human ovarian carcinomas. *Genes Chromosomes Cancer* **20**: 234–242

Conejo JR, Kleeff J, Koliopanos A, Matsuda K, Zhu ZW, Goecke H, Bicheng N, Zimmermann A, Korc M, Friess H and Buchler MW (2000) Syndecan-1 expression is up-regulated in panceatic but not in other gastrointestinal cancers. *Int J Cancer* **88**: 12–20

Dhodapkar MV, Abe E, Theus A, Lacy M, Langford JK, Barlogie B and Sanderson RD (1998) Syndecan-1 is a multifunctional regulator of myeloma pathobiology: control of tumor cell survival, growth, and bone cell differentiation. *Blood* **91**(8): 2679–88

Esko J (1999) Genetic analysis of proteoglycan structure, function and assembly. In *Cell Surface Proteoglycans in Signalling and Development*, Lander A, Nakato H, Selleck SB, Turnbull JE and Coath C (ed) pp 35–40. HFSP: Strasbourg

Gallagher JT and Lyon M (2000) Heparan sulphate: Molecular structure and interactions with growth factors and morphogens. In: *Proteoglycans: Structure, Biology and Molecular Interactions* (Ed. Iozzo, RV), Marcel Dekker, New York

Gonzalez AD, Kaya M, Shi W, Song H, Testa JR, Penn LZ and Filmus J (1998) OCI-5/GPC3, a glypican encoded by a gene that is mutated in the Simpson-Golabi-Behmel overgrowth syndrome, induces apoptosis in a cell line-specific manner. *J Cell Biol* **141**: 1407–1414

Hecht JT, Hogue D, Strong LC, Hansen MF, Blanton SH and Wagner M (1995) Hereditary multiple exostosis and chondrosarcoma: linkage to chromosome 11 and loss of heterozygosity for EXT-linked markers on chromosomes 11 and 8. *Am J Hum Genet* **56**: 1125–1131

Hennekam RCM (1991) Hereditary multiple exostosis. *J Med Genet* **28**: 262–266

Hirabayashi K, Numa F, Suminami Y, Murakami A, Murakami T and Kato H (1998) Altered proliferative and metastatic potential associated with increased expression of syndecan 1 *Tumour Biol* **19**: 454–463

Inki P and Jalkanen M (1996) The role of syndecan-1 in malignancies. *Ann Med* **28**: 63–67

Jayson GC, Lyon M, Paraskeva C, Turnbull JE, Deakin JA and Gallagher JT (1998) Heparan sulfate undergoes specific structural changes during the progression from human colon adenoma to carcinoma in vitro. *J Biol Chem* **273**(1): 51–7

Kitagawa H, Shimakawa H and Sugahara K (1999) The tumor suppressor EXT-like gene EXTL2 encodes an α1, 4-N-Acetylhexosaminyltransferase that transfers N-acetylgalactosamine and N-Acetylglucosamine to the common glycosaminoglycan-protein linkage region. *J Biol Chem* **274**: 13933–13937

Kleef J, Ishiwata T, Kumbasar A, Friess H, Buchler MW, Lander AD and Korc M (1998) The cell-surface heparan sulphate proteoglycan glypican-1 regulates growth factor action in pancreatic carcinoma cells and is overexpressed in human pancreatic cancer. *J Clin Invest* **102**(9): 1662–1673

Kumar-Singh S, Jacobs W, Dhaene K, Weyn B, Bogers J, Weyler J and Van Marck E (1998) Syndecan-1 expression in malignant mesothelioma: corrlelation with cell differentiation, WT1 expression, and clinical outcome. *J Pathol* **186**: 300–305

Lin H, Huber R, Schlessinger D and Morin PJ (1999) Frequent silencing of the GPC3 gene in ovarian cancer cell lines. *Cancer Res* **59**: 807–810

Lind T, Tufaro F, McCormick C, Lindahl U and Lidholt K (1998) The putative tumor suppressors EXT1 and EXT2 are glycosyltransferases required for the biosynthesis of heparan sulfate. *J Biol Chem* **273**: 26265–26268

Lindahl U, Kusche-Gullberg M and Kjellen L (1998) Regulated diversity of heparan sulfate. *J Biol Chem* **273**(39): 24979–82

Matsumoto A, Ono M, Fujimoto Y, Gallo L, Bernfield M and Kohgo Y (1997) Reduced expression of Syndecan 1 in human hepatocellular carcinoma with high metastatic potential *Int J Cancer* **74**: 482–491

McCormick C, Leduc Y, Martindale D, Mattison K, Esford LE, Dyer AP and Tufaro F (1998) The putative tumour suppressor EXT1 alters the expression of cell-surface heparan sulfate *Nat Genet* **19**: 158–161

McKenzie E, Tyson K, Stamps A, Smith P, Turner P, Barry R, Hircock M, Patel S, Barry E, Stubberfield C, Terrett J and Page M (2000) Cloning and expression profiling of Hpa2, a novel mammalian heparanase family member. *Biochem Biophys Res Commun* **276**: 1170–1177

Merry CL, Lyon M, Deakin JA, Hopwood JJ and Gallagher JT (1999) Highly-sensitive sequencing of the sulphated domains of heparan sulphate. *J Biol Chem* **274**: 18455–18462

Murthy SS, Shen T, De Rienzo A, Lee W-C, Ferriola PC, Jhanwar SC, Mossman BT, Filmus J and Testa JR (2000) Expression of GPC3, an X-linked recessive overgrowth gene, is silenced in malignant mesothelioma. *Oncogene* **19**: 410–416

Nackaerts K, Verbeken E, Deneffe G, Vanderschueren B, Demedts M and David G (1997) Heparan sulphate proteoglycan expression in human lung cells. *Int J Cancer* **74**: 335–345

Paine-Saunders S, Viviano BL, Zupich J, Skarnes WC and Saunders S (2000) Glypican-3 controls cellular responses to Bmp4 in limb patterning and skeletal development. *Dev Biol* **225**: 179–187

Pilia G, Hughes-Benzie RM, MacKenzie A, Baybayan P, Chen EY, Huber R, Neri G, Cao A, Forabosco A and Schlessinger D (1996) Mutations in GPC3, a glypican gene, cause the Simpson-Golabi -Behmel overgrowth syndrome. *Nat Genet* **12**: 241–247

Pye DA, Vives RR, Turnbull JT, Hyde P and Gallagher JT (1998) Heparan sulfate oligosaccharides require 6-O-sulfation for promotion of basic fibroblast growth factor mitogenic activity. *J Biol Chem* **273**: 22936–22942

Raskind WH, Conrad EU, Chansky H and Matsushita M (1995) Loss of heterozygosity in chondrosarcomas for markers linked to hereditary multiple exostoses loci on chromosomes 8 and 11. *Am J Hum Genet* **56**: 1132–1139

Seidel C, Sundan A, Hjorth M, Turesson I, Dahl IM, Abildgaard N, Waage A and Borset M (2000) Serum syndecan-1: a new independent prognostic marker in multiple myeloma. *Blood* **95**(2): 388–92

Selleck SB (2000) Proteoglycans and pattern formation – Sugar biochemistry meets developmental genetics. *Trends Genet* **16**: 206–212

Sharma B, Handler M, Eichstetter I, Whitelock JM, Nugent MA and Iozzo RV (1998) Antisense targetting of perlecan blocks tumor growth and angiogenesis in vivo. *J Clin Invest* **102**(8): 1599–1608

Stanley MJ, Stanley MW, Sanderson RD and Zera R (1999) Syndecan-1 expression is induced in the stroma of infiltrating breast carcinoma. *Am J Clin Pathol* **112**: 377–383

Stickens D, Brown D and Evans G (2000) EXT genes are differentially expressed in bone and cartilage during mouse embryogenesis. *Dev Dyn* **218**: 452–464

Van Kuppevelt TH, Dennissen MABA, van Venrooij WJ, Hoet RMA and Veerkamp JH (1998) Generation and application of type-specific anti-heparan sulfate antibodies using phage display technology. *J Biol Chem* **273**: 12960–12966

Veugelers M, Cat BD, Muyldermans SY, Reekmans G, Delande N, Frints S, Legine E, Fryns JP, Schrander-Stumpel C, Weidle B, Magdalena N and David G (2000) Mutational analysis of the GPC3/GPC4 glypican gene cluster on Xq26 in patients with Simpson-Golabi-Behmel syndrome: identification of loss-of-function mutations in the GPC3 gene. *Hum Mol Genet* **9**: 1321–8

Vlodavsky I, Friedmann Y, Elkin M, Aingorn H, Atzmon R, Ishai-Michaeli R, Bitan M, Pappo O, Peretz T, Michal I, Spector L and Pecker I (1999) Mammalian heparanase: Gene cloning, expression and function in tumor progression and metastasis. *Nat Med* **5**: 793–802

 *© 2001 Cancer Research Campaign*

## References

[PDF_00434] Alexander C. M., Reichsman F., Hinkes M. T., Lincecum J., Becker K. A., Cumberledge S., Bernfield M. (2000). Syndecan-1 is required for Wnt-1-induced mammary tumorigenesis in mice.. Nat Genet.

[PDF_00437] Arai T., Akiyama Y., Nagasaki H., Murase N., Okabe S., Ikeuchi T., Saito K., Iwai T., Yuasa Y. (1999). EXTL3/EXTR1 alterations in colorectal cancer cell lines.. Int J Oncol.

[PDF_00440] Aviezer D., Iozzo R. V., Noonan D. M., Yayon A. (1997). Suppression of autocrine and paracrine functions of basic fibroblast growth factor by stable expression of perlecan antisense cDNA.. Mol Cell Biol.

[PDF_00443] Bernfield M., Götte M., Park P. W., Reizes O., Fitzgerald M. L., Lincecum J., Zako M. (1999). Functions of cell surface heparan sulfate proteoglycans.. Annu Rev Biochem.

[PDF_00446] Chang Z., Meyer K., Rapraeger A. C., Friedl A. (2000). Differential ability of heparan sulfate proteoglycans to assemble the fibroblast growth factor receptor complex in situ.. FASEB J.

[PDF_00449] Choi C., Cho S., Horikawa I., Berchuck A., Wang N., Cedrone E., Jhung S. W., Lee J. B., Kerr J., Chenevix-Trench G. (1997). Loss of heterozygosity at chromosome segment Xq25-26.1 in advanced human ovarian carcinomas.. Genes Chromosomes Cancer.

[PDF_00453] Conejo J. R., Kleeff J., Koliopanos A., Matsuda K., Zhu Z. W., Goecke H., Bicheng N., Zimmermann A., Korc M., Friess H. (2000). Syndecan-1 expression is up-regulated in pancreatic but not in other gastrointestinal cancers.. Int J Cancer.

[PDF_00457] Dhodapkar M. V., Abe E., Theus A., Lacy M., Langford J. K., Barlogie B., Sanderson R. D. (1998). Syndecan-1 is a multifunctional regulator of myeloma pathobiology: control of tumor cell survival, growth, and bone cell differentiation.. Blood.

[PDF_00467] Gonzalez A. D., Kaya M., Shi W., Song H., Testa J. R., Penn L. Z., Filmus J. (1998). OCI-5/GPC3, a glypican encoded by a gene that is mutated in the Simpson-Golabi-Behmel overgrowth syndrome, induces apoptosis in a cell line-specific manner.. J Cell Biol.

[PDF_00471] Hecht J. T., Hogue D., Strong L. C., Hansen M. F., Blanton S. H., Wagner M. (1995). Hereditary multiple exostosis and chondrosarcoma: linkage to chromosome II and loss of heterozygosity for EXT-linked markers on chromosomes II and 8.. Am J Hum Genet.

[PDF_00475] Hennekam R. C. (1991). Hereditary multiple exostoses.. J Med Genet.

[PDF_00481] Jayson G. C., Lyon M., Paraskeva C., Turnbull J. E., Deakin J. A., Gallagher J. T. (1998). Heparan sulfate undergoes specific structural changes during the progression from human colon adenoma to carcinoma in vitro.. J Biol Chem.

[PDF_00484] Kitagawa H., Shimakawa H., Sugahara K. (1999). The tumor suppressor EXT-like gene EXTL2 encodes an alpha1, 4-N-acetylhexosaminyltransferase that transfers N-acetylgalactosamine and N-acetylglucosamine to the common glycosaminoglycan-protein linkage region. The key enzyme for the chain initiation of heparan sulfate.. J Biol Chem.

[PDF_00488] Kleeff J., Ishiwata T., Kumbasar A., Friess H., Büchler M. W., Lander A. D., Korc M. (1998). The cell-surface heparan sulfate proteoglycan glypican-1 regulates growth factor action in pancreatic carcinoma cells and is overexpressed in human pancreatic cancer.. J Clin Invest.

[PDF_00492] Kumar-Singh S., Jacobs W., Dhaene K., Weyn B., Bogers J., Weyler J., Van Marck E. (1998). Syndecan-1 expression in malignant mesothelioma: correlation with cell differentiation, WT1 expression, and clinical outcome.. J Pathol.

[PDF_00495] Lin H., Huber R., Schlessinger D., Morin P. J. (1999). Frequent silencing of the GPC3 gene in ovarian cancer cell lines.. Cancer Res.

[PDF_00497] Lind T., Tufaro F., McCormick C., Lindahl U., Lidholt K. (1998). The putative tumor suppressors EXT1 and EXT2 are glycosyltransferases required for the biosynthesis of heparan sulfate.. J Biol Chem.

[PDF_00500] Lindahl U., Kusche-Gullberg M., Kjellén L. (1998). Regulated diversity of heparan sulfate.. J Biol Chem.

[PDF_00502] Matsumoto A., Ono M., Fujimoto Y., Gallo R. L., Bernfield M., Kohgo Y. (1997). Reduced expression of syndecan-1 in human hepatocellular carcinoma with high metastatic potential.. Int J Cancer.

[PDF_00505] McCormick C., Leduc Y., Martindale D., Mattison K., Esford L. E., Dyer A. P., Tufaro F. (1998). The putative tumour suppressor EXT1 alters the expression of cell-surface heparan sulfate.. Nat Genet.

[PDF_00508] McKenzie E., Tyson K., Stamps A., Smith P., Turner P., Barry R., Hircock M., Patel S., Barry E., Stubberfield C. (2000). Cloning and expression profiling of Hpa2, a novel mammalian heparanase family member.. Biochem Biophys Res Commun.

[PDF_00512] Merry C. L., Lyon M., Deakin J. A., Hopwood J. J., Gallagher J. T. (1999). Highly sensitive sequencing of the sulfated domains of heparan sulfate.. J Biol Chem.

[PDF_00515] Murthy S. S., Shen T., De Rienzo A., Lee W. C., Ferriola P. C., Jhanwar S. C., Mossman B. T., Filmus J., Testa J. R. (2000). Expression of GPC3, an X-linked recessive overgrowth gene, is silenced in malignant mesothelioma.. Oncogene.

[PDF_00519] Nackaerts K., Verbeken E., Deneffe G., Vanderschueren B., Demedts M., David G. (1997). Heparan sulfate proteoglycan expression in human lung-cancer cells.. Int J Cancer.

[PDF_00522] Paine-Saunders S., Viviano B. L., Zupicich J., Skarnes W. C., Saunders S. (2000). glypican-3 controls cellular responses to Bmp4 in limb patterning and skeletal development.. Dev Biol.

[PDF_00525] Pilia G., Hughes-Benzie R. M., MacKenzie A., Baybayan P., Chen E. Y., Huber R., Neri G., Cao A., Forabosco A., Schlessinger D. (1996). Mutations in GPC3, a glypican gene, cause the Simpson-Golabi-Behmel overgrowth syndrome.. Nat Genet.

[PDF_00529] Pye D. A., Vives R. R., Turnbull J. E., Hyde P., Gallagher J. T. (1998). Heparan sulfate oligosaccharides require 6-O-sulfation for promotion of basic fibroblast growth factor mitogenic activity.. J Biol Chem.

[PDF_00532] Raskind W. H., Conrad E. U., Chansky H., Matsushita M. (1995). Loss of heterozygosity in chondrosarcomas for markers linked to hereditary multiple exostoses loci on chromosomes 8 and 11.. Am J Hum Genet.

[PDF_00535] Seidel C., Sundan A., Hjorth M., Turesson I., Dahl I. M., Abildgaard N., Waage A., Borset M. (2000). Serum syndecan-1: a new independent prognostic marker in multiple myeloma.. Blood.

[PDF_00538] Selleck S. B. (2000). Proteoglycans and pattern formation: sugar biochemistry meets developmental genetics.. Trends Genet.

[PDF_00540] Sharma B., Handler M., Eichstetter I., Whitelock J. M., Nugent M. A., Iozzo R. V. (1998). Antisense targeting of perlecan blocks tumor growth and angiogenesis in vivo.. J Clin Invest.

[PDF_00543] Stanley M. J., Stanley M. W., Sanderson R. D., Zera R. (1999). Syndecan-1 expression is induced in the stroma of infiltrating breast carcinoma.. Am J Clin Pathol.

[PDF_00546] Stickens D., Brown D., Evans G. A. (2000). EXT genes are differentially expressed in bone and cartilage during mouse embryogenesis.. Dev Dyn.

[PDF_00551] Veugelers M., Cat B. D., Muyldermans S. Y., Reekmans G., Delande N., Frints S., Legius E., Fryns J. P., Schrander-Stumpel C., Weidle B. (2000). Mutational analysis of the GPC3/GPC4 glypican gene cluster on Xq26 in patients with Simpson-Golabi-Behmel syndrome: identification of loss-of-function mutations in the GPC3 gene.. Hum Mol Genet.

[PDF_00556] Vlodavsky I., Friedmann Y., Elkin M., Aingorn H., Atzmon R., Ishai-Michaeli R., Bitan M., Pappo O., Peretz T., Michal I. (1999). Mammalian heparanase: gene cloning, expression and function in tumor progression and metastasis.. Nat Med.

[PDF_00548] van Kuppevelt T. H., Dennissen M. A., van Venrooij W. J., Hoet R. M., Veerkamp J. H. (1998). Generation and application of type-specific anti-heparan sulfate antibodies using phage display technology. Further evidence for heparan sulfate heterogeneity in the kidney.. J Biol Chem.

